# Intake of Gnetum Africanum and Dacryodes Edulis, Imbalance of Oxidant/Antioxidant Status and Prevalence of Diabetic Retinopathy in Central Africans

**DOI:** 10.1371/journal.pone.0049411

**Published:** 2012-12-03

**Authors:** Mvitu-Muaka Moise, Longo-Mbenza Benjamin, Mokondjimobe Etienne, Gombet Thierry, Kibokela Ndembe Dalida, Tulomba Mona Doris, Wayiza Masamba Samy

**Affiliations:** 1 Department of Ophthalmology, University of Kinshasa, Kinshasa, The Republic of Congo; 2 Faculty of Health Sciences, Walter Sisulu University, Mthatha, South Africa; 3 Faculty of Health Sciences, University of Marien Ngouabi, Brazzaville, The Republic of Congo; 4 Department of Neuropsychiatry, University of Kinshasa, The Republic of Congo; 5 Biostatistics Unit, Lomo Medical Center and Heart of Africa Center of Cardiology, Kinshasa, The Republic of Congo; 6 Department of Chemistry and Chemical Technology, Walter Sisulu University, Mthatha, South Africa; University of Colorado Denver, United States of America

## Abstract

**Objective:**

To estimate the prevalence of DR and to correlate cardiometabolic, sociodemographic, and oxidant/antioxidant imbalance data to the prevalence of DR.

**Design:**

This case-control study included type 2 DM (T2 DM) patients with DR (n = 66), T2 DM patients without DR (N = 84), and healthy controls (n = 45) without DR, in Kinshasa town. Diet, albuminemia, serum vitamins, and 8-isoprostane were examined.

**Results:**

No intake of safou (OR = 2.7 95% CI 1.2–5.8; P = 0.014), low serum albumin <4.5 g/dL (OR-2.9 95% CI 1.4–5.9; P = 0.003), no intake of fumbwa (OR = 2.8 95% CI 1.2–6.5; P = 0.014), high 8-isoprostane (OR = 14.3 95% CI 4.5–46; P<0.0001), DM duration ≥5 years (OR = 3.8 95% CI 1.6–9.1; P = 0.003), and low serum vitamin C (OR = 4.5 95% CI 1.3–15.5; P = 0.016) were identified as the significant independent determinants of DR.

**Conclusion:**

The important role of oxidant/antioxidant status imbalance and diet is demonstrated in DR.

## Introduction

Diabetic retinopathy (DR) is a progressive and long-term microvascular complication of diabetes mellitus (DM) defined by defects in insulin metabolism and dysfunction in carbohydrate, lipid and protein metabolism. The burden of DM is one of the most current challenging health problems in Democratic Republic of Congo (DRC) [1], [2], our Country located in Central Africa. With a prevalence rate of 31.6% in the tertiary hospital of the city of Kinshasa [3], capital of DRC (Central region of Africa), DR is the commonest microvascular complication of DM and seems to be one of leading causes of visual disability including visual impairment and blindness [4]. In this environment, pregnancy, family history of DM, longer DM duration and higher pulse pressure (arterial stiffness) are the important and independent risk factors of DR [3].

The preventive role of intake of vegetables rich in antioxidants, control of chronic hyperglycemia, early detection of microalbuminuria, control of dyslipidemia, control of arterial hypertension and vitamins supplements against metabolic syndrome (MetS), type 2 diabetes mellitus (T2DM) and diabetic complications is now reported within sub-Saharan Africa and outside Africa [5]–[8]. African pear safou (Dacryodes edulis) [9] and fumbwa vegetables (GNETUM AFRICANUM), popular food plants in tropical Africa, are well known for their high concentrations in antioxidants [5].

In DM, chronic hyperglycemia induces free radicals as mediators of diabetic complications such as DR [9]–[11]. These reactive oxygen species (ROS) react with biomolecules such as lipids, carbohydrates, proteins, nucleic acids, and macromolecules of connective tissue [12]. With advanced nutrition transition [13], people in Kinshasa are prone to excessive intake of alcohol, unsaturated fat, refined sugars and salt, but lack or very low intake of fibers, fruits and vegetables [14]. Fruits and vegetables contain vitamins, fibers and micronutrients which are supposed to prevent DR by affecting protein glycosylation, insulin sensitivity, retinal blood flow, and oxidative stress [15]–[19].

However, no significant associations were observed between serum levels of major dietary antioxidants and DR in United States of America [20]. Furthermore, the findings from certain intervention studies with antioxidants are elusive or unsucceful, and render the role of oxidative stress in DM questionable [21].

Up to now, there are no studies in Central Africa dealing with available oxidative stress biomarkers acting by Lipid Peroxidation (8-isoprostane, TBARS, Oxidized low-density lipoprotein), DNA Oxidation (8-hydroxy-deoxyguanosine), initiation of the breakdown of extracellular glutathione (gamma-glutamyl transferase), non-enzymatic antioxidants (total antioxidant status, uric acid, glutathione, vitamin C, vitamin E, vitamin D, selenium, zinc, magnesium, albumin), and enzymatic antioxidants (superoxide dismutase, glutathione peroxidase), and vegetables-fruits intake.

The aim of this study was to determine the relationship between cardiometabolic factors, sociodemographic factors, dietary intake, lifestyle changes, DM characteristics and oxidative stress/antioxidant biomarkers with the presence of DR in Bantu people from Central region of Africa. The outcome of the study may lead to a possible DR preventive effect of African antioxidants rich foods by reducing oxidative stress or lipid peroxidation.

## Materials and Methods

This survey was conducted in Kinshasa city, DRC, from July to September 2010, and is a part of the epidemiologic approach of new and established risk factors for T2DM and diabetic complications in the community [1]–[5], [14]. The study population was selected as a probability sample of 150 T2DM patients aged ≥20 years and drawn from the list of 1500 diabetics managed in each Kinshasa District. Kinshasa city enjoys a tropical climate with 7 million inhabitants [22]. Classification of rural Lukunga and Tshangu districts vs. urban Funa and Mont Amba districts was based on comprehensive development criteria defined by the Congolese government [23]. The cases were matched for sex and age to 45 apparently healthy and non-diabetic people recruited from public places of each Kinshasa district (party lounges). Interviews, clinical examination and eye examination were performed at Saint Joseph Hospital Division of Ophthalmology in Kinshasa Limete, DRC.

T2 DM was defined as a fasting serum glucose ≥126 mg/dL (≥7.o mmol/L), the use of anti-diabetic agents (International Statistical Classification of Diseases, 10^th^ Revision, E11) according to the Expert Committee of the Diagnosis of DM [24]. Cases with fever, cancer, chronic infection, antioxidants supplements, lipid lowering treatment and refusal to participate in the study were excluded. The controls were healthy individuals without any DM. The study protocol was approved by the local Ethics Committee and was conducted according to the principles of Helsinki Declaration. All participants gave informed and written consent.

### Data Collection

#### Sociodemographic data, lifestyle and environmental exposures

Information on age, gender, rural-urban migration, residence, education level, living time in Kinshasa, and DM duration was assessed with a personal interview.

### Dietary Assessment

**Figure 1:**
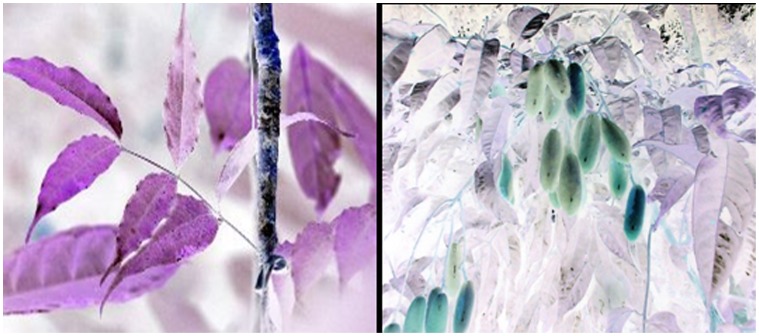
Photos of Fumbwa leaves (A) and Africanum pear Safou fruit (B).

To improve data quality and completeness, the questionnaire about dietary intake was administered by trained interviewers. All participants were asked to respond to a questionnaire concerning semi-quantitative food, frequency and intake (no vs. yes) of fumbwa vegetables and African pear safou (Figure 1), and number of portion size of other vegetables rich in antioxidants [5]. The 24-h dietary was assessed for darkly green fumbwa leaves and other vegetables intake, while vividly black, blue or pink African pear safou fruit concerned consumption during the 10 months (September 2009 to June 2010 : rainy season) before examination.

### Physical Examination

Clinical examination included measurements of current body weight, waist circumference (WC), height and blood pressure (BP). Weight in light clothes was measured to the nearest 100 g using a Soehnle scale (Soehnle-Waagen GmGh Co, Murrhardt, Germany). Height was measured to the nearest 0.5 cm using a portable locally manufactured stadiometer. Body mass index (BMI) was calculated as weight divided by height in m squared (Kg/m^2^). Three consecutive diastolic (DBP) and systolic (SBP) BP were recorded on the right arm using a standard mercury sphygmomanometer with appropriate cuff sizes at intervals longer than 2 min after 15 minutes of rest in seated participants. The average of the second and third readings was used in the present analyses. Waist circumference (WC) was measured after a gentle expiration between the lower rib margins and the iliac crest to the nearest millimeter using a flexible tape, with participants standing with their heels held together. Anthropometric measures were performed using standardized protocols.

### Laboratory Data

Blood samples were collected as per the Clinical and Laboratory Standards Institute (CLSI) document [25]–[27]. Samples were collected in one heparinized vacutainer (4 ml) and one plain vacutainer (4 ml) for each participant after a 10–12 h- overnight fasting. They were immediately analyzed at LOMO Central Laboratories, Kinshasa Limete, DRC, for cardiometabolic risk (glucose, HbA1c, Insulin, total cholesterol, triglycerides, LDL-C, HDL-C, non-HDL, VLDL, Total cholesterol/HDL ratio, triglycerides/HDL ratio, LDL/HDL ratio), certain antioxidant markers (uric acid, Albumin), and certain oxidative stress markers (gamma-glutamyl transferase, Oxidized LDL).

Fasting plasma glucose (FPG) levels were measured using glucose-oxidase method. Plasma insulin was also assayed using Mercodia kits. Enzyme activities for serum gamma-glutamyl transferase (GGT) were determined according to standard laboratory procedures. Serum total cholesterol (TC) for CHOD-PAP method, triglycerides (TG) for GPO-PAP method, high-density lipoprotein-cholesterol (HDL-C) for IRC method, uric acid, and low-density lipoprotein cholesterol (LDL-C) were measured using enzymatic colorimetric kits (Biomerieux, Marcy l’Etoile, France).

Antibodies against Oxidized LDL-cholesterol (oxLDL) were measured using solid phase two-site immunoassay based on the direct sandwich technique; there are two genetic determinants on the oxidized apoliprotein B molecules (Mercodia AB, Sylveniusgaton 8A, SE 75 450, Uppsala, Sweden). The laboratory tests in LOMO Medical Laboratories were assayed using an automatic analyzer (Hospitex Diagnostics, Florence, Italy). Glycated hemoglobin fractions were measured in fresh anticoagulated blood samples with both migration set-up using a semi-automated multi-parameter instrument (HYDRASYS system, Serbia, Evry, France) and densitometric scanning of unstained gels 9HYDRGEL 7/15 hBa1C) performed on HYRYS Densitometer and gel carrier O and specific HbA1c (NGSP) software (HYRYS densitometer, Serbia, Evry, France). After including a control blood sample into each run of samples, relative concentrations (%) of three fractions in each hemolyzate were obtained by the Densitometer scanning as follows: the most cathodic corresponding to the minor glycated hemoglobin A1c (HbA1c), and the most anodic being the main fraction containing Ao and A2 hemoglobins.

To ensure pre-analytical conditions, quality goals, storage temperature, storage time, and transport of blood samples towards South Africa (mail, courier pick-up, or brought to laboratories by express coach), we met centrifugation requirements and we separated the blood samples at LOMO Medical Laboratory within 45–60 min.

The majority of markers of oxidative stress, total antioxidant status (TAOS), micronutrients (Zinc, Selenium, Magnesium), vitamins (C, D, E), superoxide dismutase (SOD), 8-Isoprostane, 8-hydroxydeoxyguanosine (8-OHdG), erythrocyte glutathione peroxidase (GPx), and thiobarbutiric acid reacting substances (TBARS) were measured at the Analytical Chemistry Laboratory of the Walter Sisulu University, Mthatha, South Africa. Commercial Cayman’s Kits (Cayman Chemical Company, Ann Arbor, MI, USA) were used to measure TAOS, SOD, and 8-isoprostane in plasma. Serum 8-OHdG levels were measured by utilizing a competitive enzyme linked immunosorbent assay method on Biomerieux Reader version and commercial kits supplied by Northwest Laboratories (Northwest Life Science Specialties, LLC, Vancouver, WA 98662, Canada).

Levels of TBARS were measured from serum samples and expressed as mmol/L [27]. For that, samples were run simultaneously with quality control. If analysis of TBARS level varied from ±2 SD of a mean determined by a total of 60 runs, and between-week runs, the analysis was deemed unacceptable and was repeated. GSH-Px activity was measured from the washed packed cell fraction based on a kinetic method [28] and units of activity normalized per milligram of hemoglobin.

The serum Selenium (Se), Magnesium (Mag), and Zinc (Zn) concentrations were determined by direct electrothermal atomic-absorption spectrometry on AAS Unicam (Serum mineralized in microwave system, Milestone, Italy) provided selenium analysis. In sample preparation, mineralization in a microwave digestion system was used.

Serum α-tocopherol (vitamin E), ascorbic acid (vitamin C, and 1, 25 (OH) 2D (1, 25-dihydroxy-vitamin D) were analyzed using reverse phase high performance liquid chromatography (HPLC) with multiwavelength.

The very low density lipoprotein cholesterol (VLDL-C) was calculated based on the Friedewald equation [29] and excluding participants with TG ≥400 mg/dL: VLDL = TG/5.

The relative homeostasis model-based insulin resistance (HOMA-IR) was calculated using the formula of Mathews [30]: HOMA-IR  =  (fasting insulin x fasting plasma glucose)/22.5 in case of glucose concentrations in mmol/L and by 405 in case of glucose concentrations in mg/dL.

### Eye Examination

All participants in this study underwent a routine eye examination. Eye examination of each participant included visual acuity (VA) measurement, ocular alignment and motility, pupil reactivity and function, visual fields, intraocular pressure measurement (mmHg), slit lamp examination of the cornea, iris, lens and vitreous, and dilated funduscopic evaluation. This fundus examination was detailed and performed at the best possible mydriasis, after dilating the pupils with tropicamide (1%) and phenylephrine (10%), by indirect ophthalmoscopy at the slit lamp (Haag Streit 900) with 90 D lens. Participants were refracted with the use of standard subjective refraction techniques. VA was measured under similar lighting conditions by an ophthalmologist. VA was measured separately for each eye and was defined according to the lowest line on the chart for which the majority of letters were read correctly, with the full required distance correction as determined by the decimal optometric scale. Due to limited resources, retinal photography was excluded as a diagnostic tool. For the classification of DR, the modified Airlie House classification as introduced by the Early Treatment Diabetic Retinopathy Study (ETDRS) was used as follows: non-proliferative (NPDR), proliferative (PDR) and maculopathy.

### Definitions

Longer DM duration was ≥5 years (the median and quartile value). Cardiometabolic risk was defined by the variations of the mean values of age (aging), BMI, TC, 10-year absolute cardiovascular risk (10-Year ACVR) according to Framingham equation, TC/HDL ratio, Non-HDL (TC-HDL-C), TG/HDL ratio, LDL/HDL ratio, VLDL, HDL-C, LDL-C, TG, WC, SBP, DBP, and HOMA-IR.

The study groups included T2 DM with DR compared to T2 DM without DR and healthy non-diabetic individuals (controls). The presence of DR included participants with mild nonproliferative, moderately severe nonproliferative, severe nonproliferative, or proliferative, and the presence of specific diabetic lesions using the Modified Airlie House Classification scheme and the Early Treatment for Diabetic Retinopathy Study severity scale.

Imbalance of oxidant/antioxidant status was defined by increase in mean values of the assayed biomarkers of oxidative stress or their elevated levels ≥ mean values with the presence of DR, and decrease in mean values or their elevated levels ≥ mean values for the antioxidant markers with the presence of DR. The imbalance of oxidant/antioxidant status was also defined by the negative correlations between important independent determinants of oxidant and antioxidant markers of DR in all participants.

### Statistical Analyses

Data are reported as “post-hoc” proportions (%) for categorical variables and mean ± standard deviation for continuous variables. The Chi-square test was used to compare %, while comparisons of means between two groups and three groups were performed using the Student t-test and one-Way ANOVA with Bonferroni Post-Hoc test, respectively (Three possible comparisons of T2DM with DR vs. T2DM without DR; T2DM with DR vs. controls, and T2DM without DR with controls).

The univariate risk of DR was assessed in T2 DM participants by calculating Odds ratio (OR) and 95 percent confidence interval (95% CI). The multivariate analysis such as logistic binary regression model was used to assess the independent association between dietary intake, cardiometabolic risk, oxidants, antioxidants, and the presence of DR in avoiding collinearity and adjusting for the effect of potential confounding factors; enter method and Forward Wald being the consecutive strategies used to identify independent determinants.

The Pearson simple correlation coefficient r and determination coefficient R^2^ from line equation regression were used to examine the relation between identified significant independent oxidative and antioxidant markers associated with DR in all, T2 DM with DR, and T2 DM without DR.

All of the statistical analyses were two-sided and a P-value <0.05 was considered statistically significant. Data analysis was carried out using the Statistical Package for Social Sciences (SPSS) for Windows version 19 (SPSS Inc., Chicago, IL, USA).

## Results

**Figure 2:**
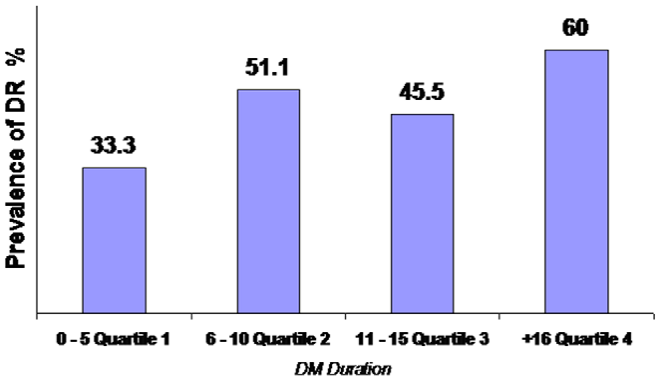
Distribution of DR across the quartiles of DM duration.

One hundred fifty T2 DM patients and 45 controls were subjected to statistical analyses. Out of all T2DM patients, 72% (n = 108) were defined by DM duration less than 10 years. Figure 2 shows that the DR cases were increasing with increasing DM duration despite 40.7% (n = 44/108) of DR cases occurred before 10 years of DM duration. In these very young adults with T2DM, the mean age in patients with DR (53.4±13.6 years) was similar (P = 0.136) to that in patients without DR (56.6±12 years).

The rate of sex, smoking, excessive alcohol intake and no intake of vegetables rich in antioxidants did not vary (P>0.05) across the 3 groups (results not shown). The demographic and cardiometabolic characteristics of participants are shown in table 1. Except for DBP, HDL-C, 10-Year ACVR, and HOMA-IR which were similar (P>0.05) across the 3 groups, the rest of the demographic and cardiometabolic characteristics varied significantly across the 3 groups. Compared with T2DM without DR, T2DM patients with DR were older and had higher BMI, DM duration, WC, SBP, TC/HDL-C ratio, TG/HDL-C ratio, LDL/HDL-C, HOMA-IR, 10-year ACVR, and also had higher levels of TC, LDL-C, HDL-C, VLDL than healthy controls. Paradoxically, T2 DM patients without DR had higher values of age, post-migration living time in Kinshasa, BMI, WC, SBP, TC, TG, TC/HDL ratio, TG/HDL ratio, LDL/HDL ratio, non-HDL-C, and VLDL compared to T2 DM patients with DR. The mean levels of age, post-migration living time in Kinshasa, WC, SBP, TC, TG, LDL-C, TC/HDL-C ratio, TG/HDL-C ratio, LDL/HDL-C ratio, non HDL-C, VLDL, 10-year ACVR, and HOMA-IR in T2DM without DR were higher than those from controls.

**Table 1:** Migration, cardiometabolic risk and DR presence.

Variables of interests	T2DM with DR n = 66	T2DM without DR n = 84	Healthy Controls n = 45	Anova, P value
Age (years)	53±13.6	56.6±12	50.7±13	0.046
Post-migration living time inKinshasa years	36±17.1	41.9±16.8	35.8±14.3	0.047
DM duration (years)	10.6±8.8	7.6±6.5	0	<0.0001
BMI (Kg/m^2^)	25.2±5	26.3±5	22.4±2.9	<0.0001
WC (cm)	93.8±16.4	95.4±12.2	81±15	<0.0001
SBP (mmHg)	128.2±22.8	129.5±20.2	120±18.7	0.040
DBP (mmHg)	78.6±12.1	78.8±13	77.6±11.9	0.854
TC (mg/dL)	200.8±58.6	205.1±54.6	174.3±41.1	0.006
TG (mg/dL)	125.2±45.6	137±45.1	75.6±7.4	<0.0001
LDL-C (mg/dL)	87.3±49.5	86.2±42.8	63.1±27	0.005
HDL-C (mg/dL)	54.4±13.7	50.9±16	56.3±14.5	0.117
TC/HDL ratio	3.8±1.3	4.3±1.6	3.2±1	<0.0001
TC/HDL-C ratio	2.6±1.3	2.8±1.5	1.4±0.4	<0.0001
LDL/HDL-C ratio	1.6±1.1	1.7±1	1.2±0.7	0.024
Non-HDL-C (mg/dL)	146.4±56.3	154.3±53.3	117.9±0.4	<0.0001
VLDL (mg/dL)	25.5±9.4	27.4±9	15.1±1.5	<0.0001
10-year ACVR	5.2±6.6	5.3±5.7	3.1±3.4	0.075
HOMA-IR	5.2±5.4	4.9±5.2	3.2±3.3	0.082

Except for uric acid and albumin which were similar (P>0.05) across the 3 groups, the mean values of the markers of oxidative stress, antioxidant status, and DM control, varied significantly across the 3 groups (Table 2). T2 DM patients with DR had higher levels of FPG, HbA1c, Ox-LDL, GGT, 8-OHdG, 8-Isoprostane, and SOD than those in T2DM without DR. T2DM patients with DR had lower levels of zinc, vitamin C, vitamin E, and vitamin D, than those from T2DM patients without DR. T2DM patients with DR had higher levels of FPG, HbA1c, Ox-LDL, GGT, 8-OHdG, 8-isoprostane, SOD, TBARS, and uric acid but lower levels of selenium, zinc, magnesium, TAOS, vitamin C, vitamin E, and vitamin D than those from controls. As shown for T2DM with DR, T2DM without DR presented significant different levels of selenium, zinc, magnesium, FPG, ox-LDL, GGT, uric acid, TBARS, SOD, 8-OHdG, 8-isoprostane, TAOS, vitamin C, vitamin E, and vitamin D in comparison with those from controls. When the study variables were made binary (any elevated vs. normal, any low vs. normal, no intake vs. yes) among all T2 DM patients, only no intake of fumbwa vegetables, no intake of African pear safou fruit, DM duration ≥5 years, high TBARS, low albumin, high SOD, high 8-OHdG, and high 8-isoprostane were significantly and univariately associated with higher risk of DR (Table 3).

**Table 2:** Markers of DM control, Biomarkers of oxidative stress and antioxidant status according to the presence of DR.

Variables of interests	T2DM with DR n = 66	T2DM without DR n = 84	Healthy Controls n = 45	Anova, P value
Selenium (µg/L)	102.7±20.4	101.2±19.4	126.4±12.1	<0.0001
Zinc (µg/L)	70.3±21.3	66.5±18.2	97.1±3.6	<0.0001
Magnesium (mmol/L)	0.7±0.3	0.7±0.3	0.8±0.1	<0.0001
FPG (mg/dL)	190.4±88.4	180.2±73.9	110.9±19.9	<0.0001
HbA1c (%)	9.8±4.3	9.3±4.1	–	<0.0001
Ox-LDL (U/L)	63.9±25.3	60.2±19.5	53.8±14.8	0.043
CGT (UI/L)	40.3±53.9	33.8±47.2	10.6±6.4	0.002
Uric acid (mg/dL)	5.4±1.8	5.4±2	4.7±1.6	0.079
TBARS (mmol)	8.7±2.5	9.1±5.2	4.4±1.4	<0.0001
SOD (U/mmol/L)	3.2±1.6	2.4±1.6	0.9±0.5	<0.0001
8-OHdG (mg/mL)	71.7±21.6	54.5±25.3	31.4±15	<0.0001
8-Isoprostane (ng/mL)	92.1±34.7	73.9±48.6	39.9±18.6	<0.0001
TAOS (mM)	1.1±0.7	1.1±0.7	1.7±0.8	<0.0001
Vitamin C (mg/dL)	1.2±0.7	2±0.6	4.5±1.5	<0.0001
Vitamin E (µmol/L)	15.9±3.6	17.4±5	28.2±3.6	<0.0001
Vitamin D (mmol/L)	25.1±14.8	38±11.3	77.3±17	<0.0001
Albumin (g/dL)	4.6±1.8	4.6±0.8	4.3±0.4	0.384

**Table 3:** Univariate risk factors of DR among T2 DM patients.

Variables of interest	Diabetic Retinopathy presence		P- value
	n (%)	OR (95% CI)	
Fumbwa vegetables intake			
No vs.	21 (60)	2.3 (1.1–5.1)	0.029
Yes	45 (39.1)		
Safou fruit intake			
No vs.	24 (60)	2.4 (1.2–5.1)	0.017
Yes	42(38.2)		
TBARS			
Elevated vs.	62 (47.3)	3.4 (1.1–10.7)	0.031
Normal	4 (21.1)		
DM duration			
<5 years	12 (26.7)		
≥5 years	54 (51.4)	2.5 (1.3–5)	0.005
Serum albumin			
Low <4.5 g/dL	44 (54.3)	2.5 (1.3–5)	0.006
Normal >4.5 g/dL	22 (31.9)		
SOD			
High vs.	60 (52.2)	5.3 (2–13.7)	<0.0001
Normal	6 (17.1)		
8-OHdG			
High vs.	62 (55.4)	10.5 (3.5–31.7)	<0.0001
Normal			

After adjusting for sex, HbA1c, TBARS, and DM duration in logistic regression model 1, only no intake of African pear safou fruit, low serum albumin, and no intake of fumbwa vegetables were identified as significant independent variables conferring each 3-fold multivariate risk of DR in these T2DM Bantu ethnic groups (Table 4).

**Table 4:** Independent determinants of DR among Central Africans with type 2 Diabetes mellitus.

	B coefficient	Standard error	Wald Chi-square	OR (95% CI)	P-value
**Independent variables**					
Safou fruit intake					
No vs. Yes	0.979	0.400	5.994	2.7 (1.2–5.8)	0.014
Serum Albumin					
Low vs. Normal level	1.061	0.362	8.594	2.9 (1.4–5.9)	0.003
Fumbwa vegetables intake					
No vs. Yes	1.035	0.423	5.992	2.8 (1.2–6.5)	
Constant	−1.342	0.330	16.549		<0.0001

However, after adjusting for migration, Ox-LDL, TBARS, GPx, and HOMA-IR, the logistic regression model 2 identified longer DM duration, low serum albumin, no intake of African pear safou fruit, and no intake of fumbwa vegetables as the independent and significant determinants of DR presence in these Bantu patients with T2 DM (Table 5).

**Table 5:** Independent determinants of DR among Central Africans with T2 DM.

	B coefficient	Standard error	Wald Chi-square	OR (95% CI)	P-value
**Independent variables**					
Serum Albumin					
Low vs. Normal level	0.921	0.372	6.129	2.5 (1.2–5.2)	0.013
DM duration					
≥5 years vs. <5 years	0.735	0.415	3.139	2.1 (0.9–4.7)	0.076
Safou fruit intake					
No vs. Yes	0.876	0.405	4.666	2.4 (1.1–5.3)	0.031
Fumbwa vegetables intake					
No vs. Yes	1.006	0.432	5.430	2.7 (1.2–6.4)	0.020
Constant	−1.022	0.370	7.645		<0.006

After adjusting for SOD, DM duration, SBP, DBP, and HbA1c, the logistic regression model3 identified only high TBARS and high 8-isoprostane as the independent and significant determinants of DR in these T2 DM patients. This multivariate risk of DR conferred by high TBARS was 4-fold (OR = 3.6 95% CI 1.1–12; P = 0.040), whereas that conferred by high 8-isoprostane was 11-fold (OR = 10.9 95% CI 3.6–33.1; P<0.0001).

After adjusting for sex, WC, SBP, DBP, SOD, 8-OHdG, migration, vitamin D, intake of other vegetables rich in antioxidants, intake of African pear safou fruit, and intake of fumbua vegetables in the logistic regression model 4, the multivariate risk of DR was multiplied by 14 times in case of high 8-isoprostane, by 5 times if avitaminosis C, by 4 times for longer DM duration, and twice by hypoalbuminemia (Table 6).

**Table 6:** Independent determinants of DR presence in T2 DM patients.

	B coefficient	Standard error	Wald Chi-square	OR (95% CI)	P-value
**Independent variables**					
8– Isoprostane					
Elevated vs. normal level	2.662	0.595	20.008	14.3 (4.5–46)	<0.0001
Serum Albumin					
Low vs. Normal level	0.871	0.402	4.699	2.4 (1.1–5.3)	0.030
DM duration					
≥5 years vs. <5 years	1.330	0.446	8.893	3.8 (1.2–13)	0.003
Serum Vitamin C					
Low vs. Normal level	1.513	0.626	5.842	4.5 (1.3–15.5)	0.016
Constant	−3.812	0.879	18.794		<0.0001

**Figure 3:**
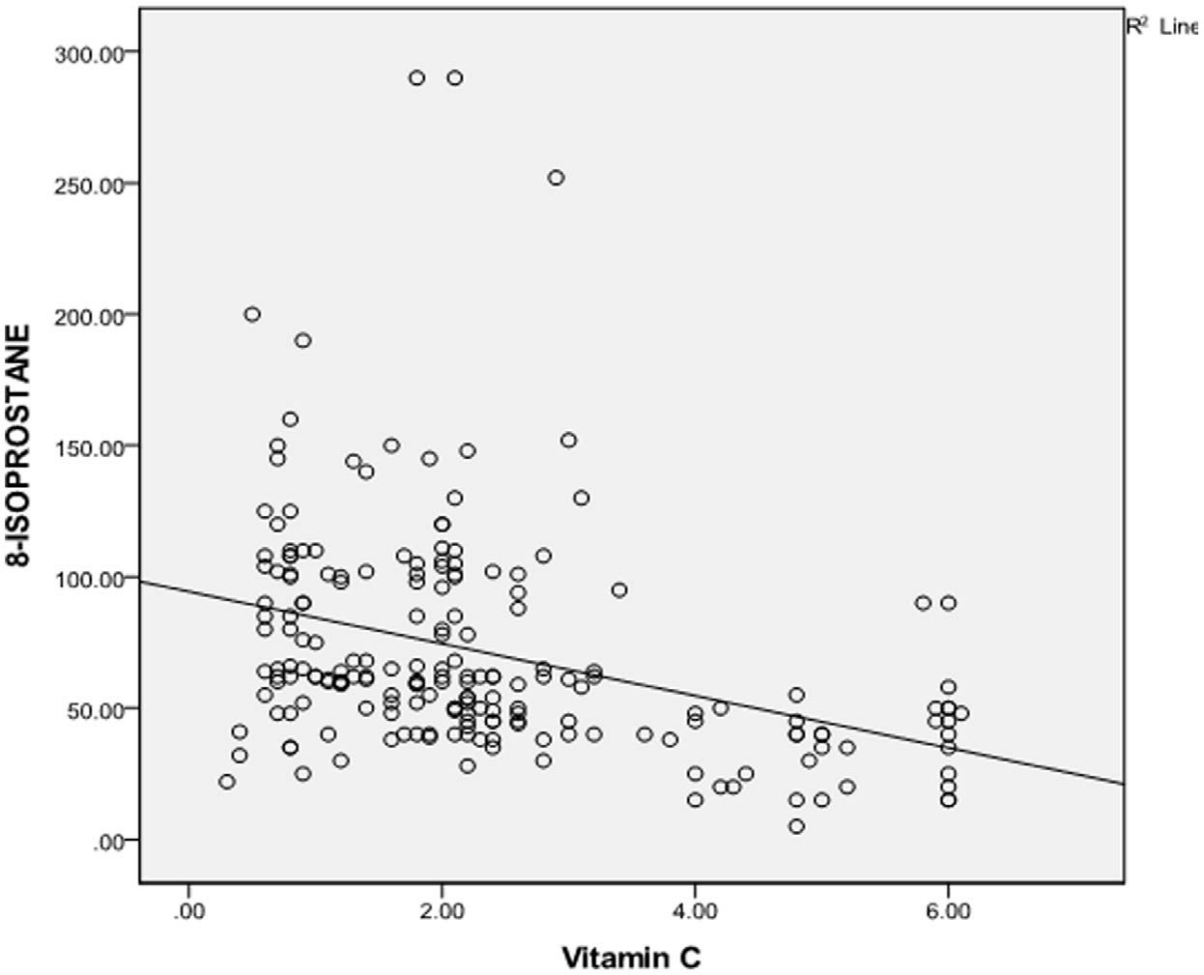
Relationship between level of 8-isoprostane and level of vitamin C in all participants.

There was a significant but negative correlation between serum 8-isoprostane and vitamin C among all T2DM patients (Figure 3).

## Discussion

In this cross-sectional analysis of Central African population-based case-control study, we confirmed the burden of DR with peculiar onset and natural history. We found significant associations of dietary intake and imbalance of oxidant/antioxidant status with DR.

The present study showed that the escalating prevalence of DR estimated 44% remains a public health problem within interval of 7–52% in Central region of Africa [3], [4], [31] and other developing African countries [32]–[35], Indian [36] and Middle-East [10] settings. However, this emerging burden of DR is still lower than the prevalence rate of DR estimated 75% in United States of America [37].

All markers of atherosclerosis, metabolic syndrome and insulin resistance were significantly higher in T2 DM patients compared to controls. The factors associated with DR and atherosclerosis were SBP, WC, and LDL-C. Many African studies report significant associations between stroke, cardiometabolic risk and DR [38]–[40]. The levels of HDL-C in T2 DM with DR were lower than the levels observed in DR patients from our previous study [39]. Smoking was uncommon as reported by other African studies [33].

Other established risk factors of DR such as urbanization after migration, control of DM, micronutrients, vitamins, antioxidant markers, and oxidative stress biomarkers were consistent with the present univariate analyses as reported by other studies [3], [5]–[8], [9]–[12], [14]–[21], [31], [33]. Paradoxically, the present study showed onset of DR in youngest individuals with shorter DM duration as often reported in Africa [3], [4], [31], [33]. The majority of DR cases in Africa occur before 10 years of DM duration, while DR cases in developed countries occur after 15–20 years of DM duration [37]. Lack of insulin therapy in poor settings [41], high and early mortality, and poor glucose control are no longer the conditions that explain exclusively the short DM duration in African DR patients [31], [33], [42], [43].

Indeed, after adjusting for traditional risk factors for DR and avoiding collinearity, multivariate analyses identified no intake of African pear safou, no intake of fumbwa leaves, low serum albumin, high TBARS, high 8-isoprostane, DM duration ≥5 years in only one logistic regression model, and vitamin C deficiency as the most important, significant and independent determinants of the presence of DR in these African patients with T2 DM.

African pear safou is a fruit produced by dacryodes edulis, a delicious, shade loving, evergreen tree and widely cultivated in tropical Africa. This edible fruit during rainy season, is a rich source of lipids, vitamins, protein, and antioxidants such as flavonoids [44]. The potential health-related functions of African Pear Safou intake include antibiosis, immunostimulation, detoxification, anti-inflammatory, antigout, antioxidant, hypoglycemic and hypolidemic properties [45], [46]. Koudou et al. [47] reported significant antioxidant activity of safou resin oil, including DPPH radical scavenging activities and inhibition of lipid peroxidation and suggesting that dacryodes edulis may help to prevent oxidative damage in the human body such as lipid peroxidation associated with cancer, premature aging, atherosclerosis and DM. Leudeu et al. [48] reported that dacryodes edulis decreases the HDL-C level in serum of rats.

Gnetum africanum locally known as fumbwa is the most popular leafy vegetable of the South-West-North parts of DRC. These leaves are rich in proteins, minerals (Na+), K, Ca, Mg, Fe and all essential aminoacids [49]. They are collected in the wild rather than cultivated throughout tropical Africa. The Asutan variety is very rich in crude fibers [50], [51].

Recent studies conducted in West Africa demonstrate a higher concentration in vitamin C from fumbwa leaves [52].

Additionally to deficiency of exogenous antioxidant supplements (lack of intake of fruits and vegetables), deficiency of endogenous antioxidants (low serum albumin and low serum vitamin C), and increased oxidative stress (high TBARS and high 8-isoprostane) may play a great role in the development of DR in these T2DM African patients. Endogenous deficient antioxidant may be enhanced by lack of intake of safou and fumbwa food plants.

Concerning antioxidant capacity, low serum albumin emerged as the most important mechanism in the development of DR. Indeed, low serum albumin reflects malnutrition-inflammatory states in itself and/or a complication of metabolic syndrome and diabetic nephropathy, which is significantly and independently associated with DR in Japanese study [53]. Furthermore, proteinuria (reflecting low serum albumin) is associated with retinol binding protein, a marker of insulin resistance recently identified as an independent risk factor of retinopathy [54]. In contrast to the findings from Millen et al. [20] and Marchioli et al. [21] without a consistent pattern of vitamins in DR prevalence or interventions, our data had shown a significant influence of low vitamin C in the presence of DR. In a synergistic action, lack of fumbwa intake (rich in fibers and vitamin C), and low serum vitamin C may impact on retinal arteriolar caliber (narrowing) and retinal venular caliber (widering) with higher risk of DR [15]). Studies report on effects of antioxidant vitamins C and E on the transient impairment of endothelium-dependent brachial artery vasoactivity following a single high-fat meal [55] as well as intrinsic nitric oxide and superoxide production regulates descending vasa recta contraction [56].Vitamin C is an important antioxidant structurally similar to glucose, capable of scavenging oxygen-derived free radicals and able to replace glucose in many chemical reactions and to prevent non-enzymatic glycosylation of proteins.

Regarding markers of oxidative stress, the results of this study indicated the importance of lipid peroxidation in DR in comparison with DNA peroxidation. Indeed high 8-OHdG, a marker of DNA peroxidation with potential mutations, was only a univariate risk factor of DR, while high TBARS and high 8-Isoprostane, both markers of lipid peroxidation were identified as risk factors of DR by both univariate and multivariate analyses in these Bantu African T2DM patients. Our data support the hypothesis of implication of lipid peroxidation, lipolysis and increase in blood free fatty acid levels in the development of DR [9], [10], [57].

Moreover, substances of membranes lipid peroxidation and other oxidants may react with SOD in DR patients [58]. Paradoxically, the increase in serum SOD was significantly associated with DR, fact explained by adaptive mechanisms.

The negative correlation between 8-isoprostane and vitamin C shown in all participants may explain the imbalance of oxidant/antioxidant status in T2DM. Thus, DR development may be accelerated by oxidative stress in relatively short T2DM duration.

T2DM, an equivalent of coronary artery disease, was characterized by higher levels of 10-year ACVR (similar levels between T2DM with DR and T2DM without DR) in comparison with controls. T2DM patients with DR had higher levels of ACVR and HOMA-IR than that from controls. These findings confirmed the significant association between DR and higher ACVR among Indians diabetics [59]. Despite their lack of independent association with DR, atherogenosis and insulin resistance may also exacerbate inflammation, endothelial dysfunction, and oxidative stress related to DR development [60].

### Clinical Implications and Perspectives for Public Health

The present results are of potential public health interest given of appropriate diet-based prevention in the face of limited medical and pharmacological resources to prevent, control, and treat cardiovascular diseases, metabolic syndrome, T2DM, and DR in Africa.

Our findings give important information on the role of vitamin C and antioxidants rich African foods [14], [61], [62] in postponing the onset of T2DM and DR. This study will emphasizes of promoting the consumption of these tropical foods which are available locally by T2DM patients as a public health measure.

Our data show a significant influence on the current management strategy for DR early detection, appropriate diet, prevention and removal of imbalance oxidant/antioxidant status.

Lack of awareness of the DR-severity related loss of vision, prevention and facilities for diagnosis, monitoring and appropriate treatment of DM must become a concern for African policy makers.

Changes in diet rich in refined sugars, salt and fat, but in vitamins, fruits and vegetables indicate a need for nutrition education, interventions, adequate processing, preservation of vegetables, and programmes on dietary diversification. Supplemental treatment based on safou fruit, fumbwa leaves, vitamins, and other antioxidants may provide new insights on oxidative stress, DR, and lead to a causal antioxidant therapy [11].

### Limitations of the Study

This study may be limited to some degree because of its cross-sectional design which is not capable to demonstrate a causal association between the majority of determinants and DR prevalence. However, 6 month-recall for safou intake may be considered as a predictor of oxidative stress.

Proteinuria, urinary creatine, and creatininemia were not measured.

### Conclusion

These findings emphasize the important role of fruit-vegetables intake and imbalance of oxidant/antioxidant status in the development of DR recommending appropriate diet rich in antioxidant supplements and tight glycemic control to postpone the onset of DR.
